# Influence of Red Corn Rich in Anthocyanins on Productive Traits, Blood Metabolic Profile, and Antioxidative Status of Fattening Lambs

**DOI:** 10.3390/ani12050612

**Published:** 2022-02-28

**Authors:** Zvonko Antunović, Josip Novoselec, Željka Klir Šalavardić, Zvonimir Steiner, Marcela Šperanda, Lidija Jakobek Barron, Mario Ronta, Valentina Pavić

**Affiliations:** 1 Faculty of Agrobiotechnical Sciences Osijek, J. J. Strossmayer University of Osijek, 31000 Osijek, Croatia; zantunovic@fazos.hr (Z.A.); jnovoselec@fazos.hr (J.N.); zsteiner@fazos.hr (Z.S.); marcela.speranda@fazos.hr (M.Š.); mronta@fazos.hr (M.R.); 2 Faculty of Food and Technology Osijek, J. J. Strossmayer University of Osijek, 31000 Osijek, Croatia; lidija.jakobek@ptfos.hr; 3 Department of Biology, J. J. Strossmayer University of Osijek, 31000 Osijek, Croatia; vpavic@biologija.unios.hr

**Keywords:** red corn, anthocyanins, lambs, productive traits, metabolic profile

## Abstract

**Simple Summary:**

In order to prevent lamb distress during weaning and avoid the occurrence of oxidative stress leading to diminished production performance, health status, or product quality, feeds rich in polyphenols (anthocyanins) are increasingly used in ruminant feeding. In the present study, lambs were allocated into three groups, with 10 lambs per group. The feed mixture for the control group (C) contained yellow corn. Lambs in experimental group I were fed feed mixtures containing yellow corn replaced by red corn at 50% (RC50). In experimental group II, red corn fully replaced (100%) yellow corn (RC100) in the lambs’ feed. The results of the present study indicate a positive effect of red corn rich in anthocyanins on the metabolic profile without any changes in the productive traits of lambs.

**Abstract:**

In this study, we aimed to evaluate the effects of different proportions of red corn rich in anthocyanins on the diet of fattening lambs considering their productive traits, blood metabolic profile, and antioxidative status. The research was carried out with 30 Merinolandschaf lambs, 90 days old and weaned. The feed mixture for lambs (n = 10) of the control group contained yellow corn, while in the feed mixture of experimental group I (n = 10), yellow corn was replaced with red corn at 50% (RC50), and in experimental group II (n = 10), yellow corn was 100% replaced with red corn (RC100). An automatic three-part differential haematology analyser was used to determine haematological parameters in whole blood, and biochemical parameters were determined in blood serum using a biochemical analyser. A diet containing red corn did not affect productive traits or the majority of the examined parameters. However, higher blood haemoglobin content, increased aspartate aminotransferase and creatine kinase activity, and decreased glucose and non-esterified fatty acids concentrations were found in the serum of RC100 lambs. These results indicate a positive effect of red corn rich in anthocyanins on the metabolic profile without any changes in the productive traits of lambs.

## 1. Introduction

The weaning period is a critical stage in the rearing of young ruminants because the organism is usually under stress, which limits growth, decreases feed intake, and impairs health status [1,2]. This is due to the still poorly developed digestive system of young animals, which also contributes to possible digestive disturbances [3]. Stress in animals may occur due to inadequate nutrition and adverse environmental effects, parameters that negatively affect the intestinal microflora, leading to the development of pathogenic microorganisms which in turn cause diarrhoea, reduce nutrient absorption [4], and induce oxidative stress. Particular importance is given to improving the adaptation of young animals to concentrate feed consumption during the suckling period as soon as possible. Therefore, various feeds enriched with polyphenols, such as anthocyanins, are increasingly used as supplements incorporated into solid feed after weaning, which could lead to reduced oxidative stress and improved immune response, health status, and meat quality in lambs [5,6]. Currently, the meat industry is trying to change the consumer’s perception by using natural compounds and plant-derived antioxidants instead of synthetic ones [7]. Improving diet composition is a key factor in improving the health status and welfare of animals [8] and enhancing livestock productivity [9,10]. Ruminants depend on microbial fermentation within the rumen to acquire energy from plant compounds. Rumen function has been manipulated by supplementing forages with readily fermentable carbohydrates and additives to improve animals’ productivity [11,12]. Logo et al. [13] pointed out that the regulatory mechanisms of anthocyanin metabolic pathways have not yet been fully clarified. There are numerous effects of anthocyanins [14], such as antioxidative, antimicrobial, anticancer, and anti-inflammatory. Rice-Evans et al. [15] reported that anthocyanins exhibit stronger antioxidant capacity than many other antioxidants. However, Jöbstl et al. [16] observed poor palatability of anthocyanins owing to bitter taste, which may cause low digestibility and lead to a negative effect on rumen fermentation and, consequently, reduced growth of animals. Abdel-Aal et al. [17] reported that most of the anthocyanins in the coloured varieties of corn are glycosylated while some of them are acylated, such as purple and scarlet corn. It is already known that anthocyanins are primarily present in red and black coloured corn varieties, and in blue corn, they are concentrated in the aleurone [18]. In general, coloured corn varieties contain between 27 and 1439 mg anthocyanins/kg of dry kernel [19,20]. In the literature [19,20], coloured corn varieties are classified according to the average total anthocyanin contents (in mg/kg dry kernel weight), which are from 99 to 379 in blue, 26.5 to 1439 in purple, 76.2 to 120 in black, and 2.5 to 696 in pink or red corn. Žilić et al. [21] reported that red corn contains 15.43 mg of anthocyanins expressed as cyanidin-3-glucoside equivalents (CGE)/kg DM, while red-yellow and dark yellow corn contain 2.50 mg and 696.07 mg of CGE/kg DM anthocyanins, respectively. There is no information available on the use of red corn in small ruminant feeding, but there are several studies on the use of purple corn [22,23,24]. The conclusions from these studies are mainly related to the positive antioxidant effects of using purple corn in diets for small ruminants.

According to these findings, we postulate that replacing yellow corn with red corn in rations for lambs will not negatively affect lambs’ performance, but could positively affect the metabolic profile due to increased concentrations of polyphenols or anthocyanins. Therefore, the aim of this paper is to find out how different proportions of red corn rich in anthocyanins affect the productive traits, blood metabolic profile, and antioxidative status of fattening lambs.

## 2. Materials and Methods

### 2.1. Experimental Design and Bioethics Standard

The research was carried out with 30 Merinolandschaf lambs during the fattening period at a family farm in Osijek-Baranya County, Croatia (45°20′05″ N; 18°18′59″ E). The post-weaning lambs were 90 days old, on average. The selected lambs were healthy and in satisfactory physical condition. Lambs were selected from ewes with single lambs. All lambs were dewormed at 2 months of age. These lambs were selected from 200 animals according to body weight, and were allotted to the control group, the experimental group with 50% red corn, and the experimental group with 100% red corn (25.01 ± 2.63, 25.04 ± 2.45, 25.07 ± 2.25 kg, respectively) before the experiment started. According to diet, the lambs were evenly divided by sex (50% male and 50% female) into three groups of 10 lambs. Each group was housed together in one pen (5 m × 4 m). An acclimatisation period was also carried out to adapt the lambs to the new feed, which lasted 7 days. The main experimental period lasted 27 days. Body condition score (BCS) was recorded using the 5-point scale according to Russel [25] (1 = thin to 5 = obese), and evaluated by a trained technician. Weighing and evaluation of BCS were carried out on the 1st and 27th days of the experiment. After the lambs’ slaughtering and bleeding, the skin was peeled off the lambs’ carcasses, and the abdominal (spleen, intestine, forestomach, stomach, and liver) and thoracic (trachea with the lungs and heart) cavity organs were removed. Afterwards, the weighing of carcasses was carried out and samples of muscle (m. semimembranosus) tissue were collected. The dressing percentage was calculated as follows: 100 × (carcass weight/live body weight). 

The trial followed the recommendations of the Animal Protection Act (NN 133/06, NN 37/13 and NN 1. kg125/13), the Legal Act on the Protection of Animals Used for Scientific Purposes (NN 55/13), the European Union Directive 2010/63/EU, and the rest of the valid legal acts related to the welfare of farm animals. Therefore, the study was approved by the Bioethics Committee for Research on Animals of the Faculty of Agrobiotechnical Sciences Osijek. 

### 2.2. Feed and Analysis of Feedstuffs

The lambs were offered the feed mixtures following their requirements (expected weight of ~32 kg) according to the National Research Council [26]. The feed mixture of lambs from the control group (C) contained only yellow corn. Yellow corn was replaced by red corn at a level of 50% in experimental group I (RC50) and at a level of 100% (RC100) in experimental group II. The red corn is an old native Croatian species. A mixture of red clover and grass hay (*Trifolium pratense* and *Lolium multiflorum*) and water were offered to lambs ad libitum. Feed mixtures were offered to the lambs twice per day. The lambs were weighed at the beginning and at the end of the study. Feed was offered at the same time each day. 

All feed samples (feed mixture, hay, yellow and red corn) were dried and then ground into a powder using a heavy metal-free ultra-centrifugal mill (ZM 200, Retsch GmbH, Haan, Germany) or knife mill (GM 200, Retsch GmbH, Haan, Germany). The feed composition was determined using the standard methods of the Association of Official Analytical Chemists [27]. The ingredients and chemical compositions of the diets are presented in Table 1. The crude protein content in the feed was estimated by the Kjeldahl method using a Kjeldahl steam distillation system (Behr, Stuttgart, Germany). The ether extract was estimated by the universal extraction system B-811 (Buchi, Flawil, Switzerland). The crude fibre content was determined by the Weende method and ME was determined according to INRAE-CIRAD-AFZ [28]. 

The extraction of polyphenols was carried out from feed samples. First, samples were weighed and 0.2 g was set in a plastic tube, then 1.5 mL of 80% (*v*/*v*) methanol was added in water. Samples were vortexed and extracted for 15 min with an ultrasonic water bath (RK 100, Berlin, Germany), and centrifuged for 10 min at 6739× *g* afterwards (Eppendorf, Hamburg, Germany). The extract was transferred into a separate plastic tube. The residue was extracted again following the same procedure using methanol (0.5 mL of 80%). These two extracts were combined to obtain a final volume of the extract of around 2 mL. The same procedure was then repeated to obtain a second and third parallel feed extract. 

The total concentration of feed polyphenols was determined following the Folin–Ciocalteu micro-method (Waterhouse). A diluted extract (20 μL) aliquot was mixed with distilled water (1580 μL) and Folin–Ciocalteu reagent (100 μL). An amount of 300 μL of sodium carbonate solution (200 g/L) was added to the mixture and shaken. After the incubation at 40 °C for 30 min in the water bath, the absorbance was read against the blank at 765 nm. Total polyphenols were expressed as mg of gallic acid equivalents (GAE)/kg of the sample weight. Data are presented as mean ± standard deviation of three parallels each measured two times. Determination of total anthocyanins and polyphenols was performed according to the method described by Jakobek et al. [29], using the Shimadzu UV-1280 spectrophotometer (Shimadzu Europe GmbH, Duisburg, Germany).

### 2.3. Blood Sampling and Analysis

Blood samples were collected from each lamb, at the beginning (1st day) and at the end (27th day) of the study, from the jugular vein (10 mL) into two sterile vacuum tubes (Venoject^®^, Sterile Terumo Europe, Leuven, Belgium) at the same time in the morning. The tubes, used for haematology analyses, contained ethylenediamine tetra-acetic acid (EDTA) as an anticoagulant. After collection, the samples were transported to the Department of Animal Production and Biotechnology (Faculty of Agrobiotechnical Sciences). The EDTA tubes were inverted several times to ensure adequate blood mixing with the anticoagulant. The automatic three-part differential haematology analyser Sysmex PocH-100iV (Sysmex Europe GmbH, Hamburg, Germany) was used to determine haematological parameters such as the number of leukocytes (WBC) and erythrocytes (RBC), the contents of haemoglobin (HGB) and haematocrit (HCT), the mean corpuscular volume (MCV), the average haemoglobin content in erythrocytes (MCH), and the mean haemoglobin concentration in erythrocytes (MCHC) in the whole blood of lambs. Afterwards, blood samples collected in sterile vacuum tubes Venoject^®^ (Sterile Terumo Europe, Leuven, Belgium) were centrifuged at 1610× *g* for 10 min and the obtained serum samples were placed into the analyser Beckman Coulter AU 400 with Total Protein Reagent (Beckman Coulter Inc., Brea, CA, USA). The serum biochemical parameter concentrations were determined, such as calcium, inorganic phosphorus, magnesium, iron, urea, glucose (GUK), total proteins (PROT), albumin (ALB), cholesterol (CHOL), LDL-cholesterol (LDL), HDL-cholesterol (HDL), triglycerides (TGC), β-hydroxybutyrate (BHB), and non-esterified fatty acids (NEFA). The activities of enzymes, such as alanine aminotransferase (ALT), aspartate aminotransferase (AST), alkaline phosphatase (ALP), creatine kinase (CK), γ-glutamyl transferase (GGT), and glutathione reductase (GR), were also determined in serum. All of the above-mentioned biochemical variables were determined using Beckman Coulter reagents (Beckman Coulter Inc., Brea, CA, USA), apart from BHB concentration, which was determined by using RANBUT (Randox Laboratories Ltd., Crumlin, UK), and NEFA, determined by a NEFA kit (Randox Laboratories Ltd., Crumlin, UK). Globulin content (GLOB) was calculated as the difference between total protein and albumin. The activities of glutathione peroxidase (GPx) and superoxide dismutase (SOD) in the serum were determined using a Ransel^®^ kit (Randox Laboratories Ltd., Crumlin, UK) and Ransod^®^ kit (Randox Laboratories Ltd., Crumlin, UK), respectively, which were analysed by an automatic Beckman Coulter AU 400 analyser (Beckman Coulter Inc., Brea, CA, USA). 

### 2.4. Measurements of Lipid Peroxidation and Antioxidative Activity of Meat and Serum

Immediately after slaughter, fresh lamb meat samples were collected from the right side of the m. semimembranosus, and visible fat was removed. The lambs’ muscle homogenates were prepared (10% *w*/*v*) in 0.05 M phosphate buffer (pH 7) using an Ultra Turrax (IKA T18 Basic, Labortechnik, Staufen, Germany) homogeniser and centrifuged at 12,000× *g* for 60 min at 4 °C. The blood was allowed to clot, and then the serum was separated immediately by centrifugation. The meat extraction and the serum dilution preparation were carried out in triplicate. The supernatant obtained was used for the measurement of DPPH radical scavenging activity and TBARS. 

#### 2.4.1. 2,2-Diphenyl-1-picrylhydrazyl (DPPH) Radical Scavenging Activity

The total antioxidant activities of meat and serum extracts were determined using the DPPH radical scavenging assay described by Qwele et al. [30], with some modifications. The serum samples were diluted (25 μL/mL) and mixed with 0.2 mM DPPH radical solution. The muscle extracts were diluted (0.01 g mL^−1^) and mixed with 0.2 mM DPPH radical solution. Ascorbic acid (AA) was used as a reference compound. All measurements were performed in triplicate. The absorbance was measured at 517 nm using a spectrophotometer (Lambda 25, PerkinElmer, MA, USA), and DPPH scavenging activity was determined using Equation (1):

DPPH activity = (A_b_ + A_s_) − A_m_)/A_b_ × 100 (1)

where A_b_ is the absorbance of 0.1 mM DPPH radical solution at λ = 517 nm, A_s_ is the absorbance of 0.1 mM extraction solution at λ = 517 nm, and A_m_ is the absorbance of 0.1 mM solution mixture of tested serum or extracts with DPPH radical at 517 nm.

#### 2.4.2. Thiobarbituric Acid Reactive Substances (TBARS) 

Lipid peroxidation in the meat and serum samples was estimated using the TBARS method according to Liu et al. [31], with slight modifications. A spectrophotometer (Lambda 25, PerkinElmer, MA, USA) was used to measure absorbances (532 nm and 600 nm). The molar extinction coefficient of malondialdehyde (MDA; 156,000 M^−1^ cm^−1^) was used to calculate the MDA concentration. The results were reported as mg of MDA equivalents per kg of meat sample and as nmoles of MDA equivalents per mL of blood serum. 

### 2.5. Statistical Analyses 

The normality of data distribution was checked by the Shapiro–Wilk test (PROC UNIVARIATE of SAS) [32]. Results are expressed as mean values and the standard error of mean estimated by the MEANS procedure of SAS [32], while the effects of treatment (C-control group; RC50-50% of red corn; RC100-100% of red corn) were analysed by the GLM procedure. Tukey’s test and differences between groups were declared significant at *p* < 0.05. Non-parametric data (body weight, WBC, MCV, NEFA, BHB, ALP, SOD, GPx, DPPH in blood on 27th day of study) were analysed with the Kruskal–Wallis H test. 

## 3. Results

Table 2 shows the production traits of lambs (body weight, average daily gain, carcass dressing, and body condition scores) fed with different proportions of red corn. 

No significant differences were found in the productive traits of the lambs (body weight and daily gain) or in the BCS of the lambs and the carcass dressing among experimental diets (Table 2). In addition, Table 3 reports a significant increase only in blood HGB content in the RC50 and RC100 lambs compared to group C at the end of the study, while other indicators did not vary significantly among the feeding treatments.

There were no significant differences in most biochemical parameters in the blood serum of lambs fed RC50 and RC100, except for glucose and NEFA concentrations (Table 4). The blood glucose and NEFA concentrations of RC100 lambs were significantly reduced at the end of the study compared to group C. 

The analysis of enzyme activity in the blood serum of lambs fed with different RC proportions revealed a significant effect, especially a significant increase in CK and AST activity in RC100 lambs compared to the C group (Table 5). Other determined enzyme activities did not differ significantly among the different dietary treatments.

The analysis of antioxidant status reveals no significant differences in TBARS or DPPH in blood serum or in the meat samples of lambs regardless of dietary treatment (Table 6).

## 4. Discussion

The weaning period represents a stressful condition for animals. In particular, in ruminants, weaning is not just a single event but a period in which milk is progressively replaced by forage and concentrate or by grain-based diets. Lambs are not ruminants at birth, and during the suckling period, the animal’s forestomach is poorly developed. As dry feeds begin to be consumed, the rumen and the reticulum begin to develop and rapidly increase in size. It has been demonstrated that small ruminants are more prone to oxidative stress due to intense metabolic requirements. The appearance of oxidative stress in ruminants endangers their productivity and their health. Therefore, research on the addition of polyphenols, including anthocyanins, in the diet of small ruminants is common, given the various biological activities of polyphenols (particularly anthocyanins) in different corn varieties [33]. In the present study, there were no significant changes in the productive traits of lambs, such as body weight and daily weight gain, or in the BCS and carcass dressing among the different feeding treatments. However, it is noticeable that the expected body weight and daily gain of the lambs were achieved in all groups (Table 2). In previous works carried out with the addition of polyphenols in lambs’ diet, the body weight was not affected by grapeseed oil or grapeseed extract addition [34,35]. Kanfantaris et al. [36] concluded that the supplementation of polyphenols in lamb diets, such as grape pomace, is more effective in improving production traits during the suckling period than in weaned lambs. 

In the present study, haematological and biochemical parameters as well as parameters of enzyme activity in the lambs’ blood ranged within the reference values established for lambs reared in similar conditions [37,38,39,40]. An increase in the haemoglobin content in the blood of the lambs of the RC50 and RC100 groups compared to the C lambs was found. This may be related to anthocyanins that stabilise erythrocyte membranes and inhibit haemoglobin polymerisation [41]. It is well known that polyphenols protect against reactive oxygen species-induced haemolysis via increased red blood cell integrity associated with the inhibition of lipid peroxidation [42]. The concentration of glucose and NEFA in the blood serum of the RC100 lambs was significantly reduced at the end of the study compared to group C. Glucose concentrations in the blood serum of the lambs in the RC50 group were reduced at the end of the study, although not significantly when compared to the C group, but it was significant compared to the RC100 lambs. There was a slight numerical decrease in the blood serum CHOL and TG concentrations of the RC50 and RC100 lambs compared to the C lambs. These changes indicate the need for a longer experimental duration when changes in glucose and NEFA concentrations would be evident with certainty. Sharma et al. [43] carried out a 12-week trial with mice fed diets with a high content of fat supplemented with isocaloric white, purple, or black whole wheat. These authors discovered that body weight gain was significantly reduced in mice fed black wheat. In contrast, both black and purple varieties of wheat reduced serum cholesterol concentrations, triglycerides, and free fatty acids while restoring normal serum glucose concentrations and insulin resistance. Chen et al. [44] carried out a study with mice fed black soybean seed coat extract (BSSCE), a rich source of anthocyanins. A significant decrease in serum glucose, cholesterol, triglyceride, and NEFA and MDA concentrations was determined, as well as an increase in serum HDL-cholesterol concentration and antioxidant enzyme (SOD, GPx, and catalase) activities. The authors emphasised that cyanidin-3-O-glucoside contributed to the BSSCE-induced hypoglycaemia and hypolipidemia in type 2 diabetes mellitus. Tian et al. [45] found a significant reduction in total blood cholesterol during the 74 days of their study when feeding goat kids a diet supplemented with 0.5 g/d or 1 g/d anthocyanin-rich purple corn pigment. The activity of AST in serum, GGT, ALP, and cholesterol concentrations are used for hepatic damage diagnosis in humans and animals [46], while ALP activity and cholesterol concentrations are used to detect bile obstruction or mild and progressive liver damage. A liver enzyme such as ALT, a specific hepatocellular enzyme released after hepatocellular damage, more than GGT, is used to assess liver damage. Despite significant differences in AST and CK at the end of the present study, it can be concluded that since their activities were within the reference values [47], no damage to the liver and muscle occurred. SOD, GPx, and catalase are all antioxidant enzymes that help maintain a healthy cellular antioxidant status [48]. The absence of significant changes in antioxidant enzyme activity (SOD, GPx, GR) might be explained by the lower absorption of anthocyanin by small ruminants compared to non-ruminant animals [49]. Hosoda et al. [22] reported that one of the reasons could be a sufficient pool of non-enzymatic antioxidants since severe oxidative stress is not determined in sheep. In a study conducted with lactating sheep fed purple corn, these authors found increased plasma SOD activity. However, due to the lack of research with red corn, the present study results were compared with studies in which purple corn was used, which contains a significantly higher content of anthocyanins than red corn [21]. The anthocyanin concentration possibly was not high enough to improve the antioxidative status based on SOD, GPx, or GR. A relatively short duration of the present study also contributed to an absence of significant differences. 

## 5. Conclusions

The administration of feed mixtures containing red corn rich in anthocyanins affected neither the productive traits nor most of the haematological and biochemical indicators of blood in the lambs studied here. However, significantly higher blood HGB content and increases in serum AST and CK activity were found in the RC100 lambs, as well as decreases in the serum GUK and NEFA concentrations compared to the C group. For further research, it is necessary to start earlier in the suckling period and extend the duration of fattening, with the inclusion in the experimental model the analysis of other muscles and qualitative properties of meat in order to more comprehensively observe the response to red corn rich in anthocyanins used in lamb diets.

## Figures and Tables

**Table 1:** The ingredients and chemical composition of the feed mixture, hay, yellow corn, and red corn used in the diets for fattening Merinolandschaf lambs.

Ingredient (g/kg Feed Mixture)	Feed Mixture	Hay ^1^	Yellow Corn	Red Corn
Corn	600			
Barley	120			
Wheat flour	23			
Soybean meal (46% CP)	100			
Extruded soybean	120			
Salt	4			
Calcium carbonate	3			
Mineral–vitamin premix ^2^	30			
Chemical content (g/kg DM)				
DM	912	958	905	907
Crude protein	157	96	100	105
Crude fibre (g/kg DM)	36	343	25	23
Crude ash (g/kg DM)	30	70	13	14
EE (g/kg DM)	51	9	38	37
ME (MJ/kg DM)	12.30	7	12.70	12.70
Polyphenols (total), mg/kg *	144.77	-	179.87	298.69
Anthocyanins (total), mg/kg **	0	-	125.37	253.04

^1^ Red clover and grass hay (*Trifolium pratense* and *Lolium multiflorum*). ^2^ Mineral–vitamin premix for lambs: 8% Ca, 5% P, 9.5% Na, 2.00% Mg, 400,000 IU vitamin A, 40,000 IU vitamin D, 500 mg vitamin E, 4000 mg Zn, 2000 mg Mn, 60 mg I, 10 mg Co, 50 mg Se. CP: crude protein; DM: dry matter; EE: ether extract; ME: metabolizable energy. * Concentrations of polyphenols in RC50 and RC 100 were 513.06 and 1276.70 mg/kg, respectively. ** Concentrations of anthocyanins in RC50 and RC 100 were 217.32 and 485.40 mg/kg, respectively.

**Table 2:** Production traits and body condition scores of weaned Merinolandschaf lambs fed various amounts of red corn.

Indicator	Day	Group (Mean)			SEM	*p*-Value
		C	RC50	RC100		
Body weight (kg)	1st	24.96	25.17	25.35	0.43	
	27th	32.35	32.18	33.14	0.60	0.642
Body condition score	1st	3.64	3.49	3.38	0.06	
	27th	3.62	3.71	3.84	0.07	0.172
Daily gain (g)	1st–27th	273.78	259.63	288.52	19.01	0.835
Carcass weight (kg)	27th	16.68	16.98	17.44	0.32	0.623
Carcass dressing (%)	27th	51.61	52.80	52.73	0.47	0.530

Mean: mean value; SEM: standard error of mean; C: control (yellow corn); RC50: red corn 50%; RC100: red corn 100%.

**Table 3:** The haematological parameters of weaned Merinolandschaf lambs fed various amounts of red corn.

Indicator	Day	Group (Mean)			SEM	*p*-Value
		C	RC50	RC100		
White blood cells (×10^9^/L blood)	1st	13.09	12.68	14.78	0.81	0.653
	27th	11.04	11.53	13.26	0.68	
Red blood cells (×10^9^/L blood)	1st	9.65	10.11	10.09	0.18	0.250
	27th	9.63	10.25	9.77	0.16	
Haemoglobin (g/L blood)	1st	123.90	124.40	123.80	2.19	0.049
	27th	113.00 ^a^	124.80 ^b^	120.40 ^b^	1.57	
Hematocrit (L/L blood)	1st	0.38	0.39	0.40	0.01	0.255
	27th	0.38	0.41	0.38	0.01	
Mean corpuscular volume (fL)	1st	39.31	39.38	39.07	0.50	0.512
	27th	39.87	39.88	38.74	0.49	
Mean corpuscular haemoglobin (pg)	1st	12.94	12.33	12.30	0.23	0.651
	27th	11.76	12.42	11.49	0.41	
Mean corpuscular haemoglobin concentration (g/L blood)	1st	331.20	315.50	317.10	8.17	0.452
	27th	297.60	308.50	321.60	7.63	
Platelet blood count (×10^9^/L blood)	1st	741.00	561.70	744.10	39.41	0.747
	27th	536.10	508.00	599.10	48.43	

Mean: mean value; SEM: standard error of mean; C: control (yellow corn); RC50: red corn 50%; RC100: red corn 100%. ^a,b^ Values in rows with different letters differ significantly (*p* < 0.05).

**Table 4:** The biochemical parameters in the blood serum of weaned Merinolandschaf lambs fed various amounts of red corn.

Indicator	Day	Group (Mean)			SEM	*p*-Value
		C	RC50	RC100		
Mg (mmol/L)	1st	1.41	1.38	1.43	0.02	0.856
	27th	1.36	1.34	1.33	0.02	
Ca (mmol/L)	1st	2.62	2.50	2.55	0.05	0.103
	27th	2.61	2.74	2.65	0.03	
Fe (µmol/L)	1st	33.39	34.94	37.13	1.52	0.257
	27th	26.82	33.42	32.62	1.76	
P-inorganic (mmol/L)	1st	2.79	2.91	2.98	0.09	0.461
	27th	2.72	2.87	2.99	0.09	
Glucose (mmol/L)	1st	5.51	5.09	5.40	0.12	0.003
	27th	5.98 ^a^	5.74 ^a^	5.27 ^b^	0.09	
Urea (g/L)	1st	4.92	3.52	4.51	0.26	0.874
	27th	5.41	5.60	5.41	0.17	
Total protein (g/L)	1st	60.65	58.38	58.90	0.51	0.865
	27th	63.35	64.030	63.06	0.73	
Albumin (g/L)	1st	29.90	29.92	30.50	0.29	0.109
	27th	29.51	31.00	30.75	0.31	
Globulin (g/L)	1st	30.75	28.46	28.40	0.46	0.654
	27th	33.84	33.03	32.31	0.66	
Cholesterol (mmol/L)	1st	1.97	1.96	1.93	0.10	0.196
	27th	1.33	1.28	1.21	0.05	
Triglycerides (mmol/L)	1st	0.34	0.36	0.36	0.03	0.532
	27th	0.23	0.22	0.18	0.02	
HDL (mmol/L)	1st	1.05	1.02	1.05	0.04	0.768
	27th	0.73	0.73	0.74	0.03	
LDL (mmol/L)	1st	0.80	0.74	0.71	0.06	0.188
	27th	0.47	0.39	0.44	0.03	
NEFA (mmol/L)	1st	0.33	0.23	0.40	0.05	0.013
	27th	0.31 ^a^	0.17 ^ab^	0.08 ^b^	0.04	
Beta hydroxybutyrate (mmol/L)	1st	0.35	0.37	0.40	0.03	0.193
	27th	0.43	0.51	0.42	0.03	

Mean: mean value; SEM: standard error of mean; C: control (yellow corn); RC50: red corn 50%; RC100: red corn 100%; Mg: magnesium; Ca: calcium; Fe: iron; P-inorganic: phosphorus; NEFA: non-esterified fatty acids. ^a,b^ Values in rows with different letters differ significantly (*p* < 0.05).

**Table 5:** The activity of enzymes in the blood serum of weaned Merinolandschaf lambs fed various amounts of red corn.

Enzymes	Day	Group (Mean)			SEM	*p*-Value
		C	RC50	RC100		
Aspartate transaminase (AST, U/L)	1st	133.43	97.59	106.76	11.64	0.021
	27th	87.96 ^a^	94.30 ^ab^	104.54 ^b^	2.54	
Alanine aminotransferase (ALT, U/L)	1st	8.76	11.22	9.42	1.01	0.875
	27th	8.82	8.35	8.86	0.43	
Alkaline phosphatase (ALP, U/L)	1st	350.68	366.26	490.17	31.54	0.119
	27th	358.30	307.48	456.97	28.28	
Gamma-glutamyl transferase (GGT, U/L)	1st	96.02	77.25	76.47	5.97	0.408
	27th	79.96	81.23	74.02	2.30	
Creatine kinase (CK, U/L)	1st	160.40	274.20	184.10	21.15	0.039
	27th	119.80 ^a^	127.80 ^ab^	162.20 ^b^	7.41	
Superoxide dismutase (SOD, U/mL)	1st	0.38	0.50	0.42	0.08	0.264
	27th	0.55	0.61	0.66	0.05	
Glutathione reductase (GR, U/L)	1st	72.35	75.91	72.26	3.16	
	27th	80.44	83.65	93.18	4.07	0.429
Glutathione peroxidase (GPx, U/L)	1st	522.60	441.22	491.80	33.13	0.882
	27th	582.30	626.30	625.10	57.35	

Mean: mean value; SEM: standard error of mean; C: control (yellow corn); RC50: red corn 50%; RC100: red corn 100%. ^a,b^ Values in rows with different letters differ significantly (*p* < 0.05).

**Table 6:** Antioxidative status of lambs’ blood serum and meat.

Parameter	Day	Group (Mean)			SEM	*p*-Value
		C	RC50	RC100		
TBARS (nmol MDA/mL blood serum)	1st	0.72	0.71	0.64	0.03	
	27th	0.84	0.83	0.73	0.04	0.370
DPPH blood serum (%) *	1st	73.63	77.48	76.21	2.30	
	27th	73.07	74.33	74.20	3.17	0.983
TBARS (mg MDAeq/kg muscle)		4.21	4.10	3.70	0.17	0.445
DPPH muscle (%) **		54.10	56.84	57.08	1.01	0.433

Mean: mean value; SEM: standard error of mean; C: control (yellow corn); RC50: red corn 50%; RC100: red corn 100%; TBARS: thiobarbituric acid reactive substances. * % DPPH radical scavenging activity at final serum concentration 25 μL/mL; ** % DPPH radical scavenging activity at final muscle concentration 0.01 g/mL.

## Data Availability

The data are available upon request.

## References

[1] Antunović Z., Šperanda M., Senčić Đ., Domaćinović M., Novoselec J. (2008). Djelotvornost probiotskog pripravka “Probios 2B” u hranidbi jaradi. Krmiva.

[2] Lucianer E., da Silva A.S., Cazarotto C.J., Alba D.F., Griss L.G., Zampar A., Vedovatto M., Kessler J.D. (2019). Addition of tannin in lamb diet after weaning: Impact on performance and hematological and biochemical variables. Acta Acientiae Vet..

[3] Boas A.S.V., Arrigoni M.B., Silveira A.C., Costa C., Chardulo L.A.L. (2003). Effects of age at weaning and feed management on the production of super-young lambs. Rev. Bras. De Zootech..

[4] Hirsh D.C., Anderson N.V. (1980). Relation of normal microflora to structures and functions of the gastrointestinal tract. Veterinary Gastroenterology.

[5] Correddu F., Lunesu M.F., Buffa G., Atzori A.S., Nudda A., Battacone G., Pulina G. (2020). Can agro-industrial by-products rich in polyphenols be advantageously used in the feeding and nutrition of dairy small ruminants?. Animals.

[6] Simitzis P.E., Charismiadou M.A., Goliomytis M., Charalambous A., Ntetska I., Giamouri E., Deligeorgis S.G. (2019). Antioxidant status, meat oxidative stability and quality characteristics of lambs fed with hesperidin, naringin or alpha-tocopheryl acetate supplemented diets. J. Sci. Food Agric..

[7] Manessis G., Kalogianni A.I., Lazou T., Moschovas M., Bossis I., Gelasakis A.I. (2020). Plant-derived natural antioxidants in meat and meat products. Antioxidants.

[8] Abbate J.M., Macrì F., Capparucci F., Iaria C., Briguglio G., Cicero L., Salvo A., Arfuso F., Ieni A., Piccione G. (2020). Administration of protein hydrolysates from anchovy (*Engraulis Encrasicolus*) waste for twelve weeks decreases metabolic dysfunction-associated fatty liver disease severity in ApoE–/–Mice. Animals.

[9] Avondo M., Pagano R., Guastella A., Criscione A., di Gloria M., Valenti B., Piccione G., Pennisi P. (2009). Diet selection and milk production and composition in Girgentana goats with different αs1-casein genotype. J. Dairy Res..

[10] Monteverde V., Congiu F., Vazzana I., Dara S., di Pietro S., Piccione G. (2017). Serum lipid profile modification related to polyunsaturated fatty acid supplementation in thoroughbred horses. J. Appl. Anim. Res..

[11] Gobindram M.N.N.E., Bognanno M., Luciano G., Avondo M., Piccione G., Biondi L. (2017). The effects of barley replacement by dehydrated citrus pulp on feed intake, performance, feeding behaviour and serum metabolic indicators in lambs. Anim. Prod. Sci..

[12] Armato L., Gianesella M., Morgante M., Fiore E., Rizzo M., Giudice E., Piccione G. (2016). Rumen volatile fatty acids × dietary supplementation with live yeast and yeast cell wall in feedlot beef cattle. Acta Agric. Scand. Sect. A-Anim. Sci..

[13] Logo C., Cassani E., Petroni K., Calvenzani V., Tonelli C., Pilu R. Study of maize genotypes rich in antocyanins for human and animal nutrition. Proceedings of the Joint Meeting AGI-SIBV-SIGA.

[14] Changxing L., Chenling M., Alagawany M., Jinhua L., Dongfand D., Gaichao W., Wenyin Z., Syed S.F., Arain M.A., Saeed M. (2018). Health benefits and potential applications of anthocyanins in poultry feed industry. Worlds Poult. Sci. J..

[15] Rice-Evans C.A., Miller N.J., Bolwell P.G., Bramley P.M., Pridham J.B. (1995). The relative antioxidant activities of plant-derived polyphenolic flavonoids. Free Radic. Res..

[16] Jöbstl E., O’Connell J., Fairclough J.P., Williamson M.P. (2004). Molecular model for astringency produced by polphenol/protein interactions. Biomacromolecules.

[17] Abdel-Aal E.S.M., Young J.C., Rabalski I. (2006). Anthocyanin composition in black, blue, pink, purple, and red cereal grains. J. Agric. Food Chem..

[18] Chatham L.A., Paulsmeyer M., Juvik J.A. (2019). Prospects for economical natural colorants: Insights from maize. Theor. Appl. Genet..

[19] Salinas M.Y., Sanchez G.S., Hernandez D.R., Lobato N.R. (2005). Characterization of anthocyanin extracts from maize kernels. J. Chromatogr. Sci..

[20] Lopez-Martinez L.X., Oliart-Ros R.M., Valerio-Alfaro G., Lee C.H., Parkin K.L., Garcia H.S. (2009). Antioxidant activity, phenolic compounds and anthocyanins content of eighteen strains of Mexican maize. LWT-Food Sci. Technol..

[21] Žilić S., Serpen A., Akillioglu G., Gökmen V., Vančcetović J. (2012). Phenolic compounds, carotenoids, anthocyanins, and antioxidant capacity of colored maize (*Zea mays* L.) kernels. J. Agric. Food Chem..

[22] Hosoda K., Miyaji M., Matsuyama H., Haga S., Ishizaki H., Nonaka K. (2012). Effect of supplementation of purple pigment from anthocyanin-rich corn (*Zea mays* L.) on blood antioxidant activity and oxidation resistance in sheep. Livest. Sci..

[23] Tian X.Z., Paengkoum P., Paengkoum S., Chumpawadee S., Ban C., Sorasak T. (2019). Purple corn (*Zea mays* L.) stover silage with abundant anthocyanins transferring anthocyanin composition to the milk and increasing antioxidant status of lactating dairy goats. J. Dairy Sci..

[24] Tian X.Z., Xin H., Paengkoum P., Paengkoum S., Ban C., Sorasak T. (2019). Effects of anthocyanin-rich purple corn (*Zea mays* L.) stover silage on nutrient utilization, rumen fermentation, plasma antioxidant capacity, and mammary gland gene expression in dairy goats. J. Anim. Sci..

[25] Russel A., Boden E. (1991). Body condition scoring of sheep. Sheep and Goat Practice.

[26] National Research Council (NRC) (2007). Nutrient Requirements of Small Ruminants: Sheep, Goats, Cervids and New World Camelids.

[27] Association of Official Analytical Chemists (AOAC) (2006). Official Methods of Analysis.

[28] INRAE-CIRAD-AFZ Feed Tables Composition and Nutritive Values of Feeds for Cattle, Sheep, Goats, Pigs, Poultry, Rabbits, Horses and Salmonids. https://www.feedtables.com.

[29] Jakobek L., Matić P., Ištuk J., Barron A.R. (2021). Study of interactions between individual phenolics of aronia with barley β-glucan. Pol. J. Food Nutr. Sci..

[30] Qwele K., Hugo A., Oyedemi S.O., Moyo B., Masika P.J., Muchenje V. (2013). Chemical composition, fatty acid content and antioxidant potential of meat from goats supplemented with Moringa (*Moringa oleifera*) leaves, sunflower cake and grass hay. Meat Sci..

[31] Liu F., Dai R., Zhu J., Li X. (2010). Optimizing color and lipid stability of beef patties with a mixture design incorporating with tea catechins, carnosine, and α-tocopherol. J. Food Eng..

[32] (2012). SAS^®^ 9.4 2002.-2012.

[33] Colombo R., Ferron L., Papetti A. (2021). Colored Corn: An up-date on metabolites extraction, health implication, and potential use. Molecules.

[34] Sharifi M., Bashtani M., Naserian A.A., Farhangfar H., Rasani M., Emami A. (2019). Grape seed oil supplementation in lamb diet: Effect on meat oxidation stability and muscle fatty acids. Ital. J. Anim. Sci..

[35] Giller K., Sinza S., Messadene-Chelalib J., Marquardt S. (2021). Maternal and direct dietary polyphenol supplementation affect growth, carcass and meat quality of sheep and goats. Animal.

[36] Kafantaris I., Kotsampasi B., Christodoulou V., Makri S., Stagos D., Gerasopoulos K., Petrotos K., Goulas P., Kouretas D. (2018). Effects of dietary grape pomacesupplementation on performance, carcass traits and meat quality of lambs. In Vivo.

[37] Latimer K.S., Prasse K., Latimer K.S., Maheffey E.A., Prasse K.W. (2003). Leukocites. Duncan and Prasse’s Veterinary Laboratory Medicine—Clinical Pathology.

[38] Lepherd M., Canfield P.J., Hunt G.B., Bosward K.L. (2009). Haematological, biochemical and selected acute phase protein reference intervals for weaned female Merino lambs. Aus. Vet. J..

[39] Antunović Z., Novoselec J., Senčić Đ., Šperanda M., Steiner Z., Samac D. Production traits and blood biochemical parameters in organic lamb production. Proceedings of the 45th Croatian and 5th International Symposium on Agriculture.

[40] Antunović Z., Mioč B., Klir Šalavardić Ž., Širić I., Držaić V., Đidara M., Novoselec J. (2021). The effect of lactation stage on the hematological and biochemical parameters of the Travnik Pramenka ewes. Poljoprivreda.

[41] Sivamaruthi B.S., Kesika P., Chaiyasut C. (2020). The influence of supplementation of anthocyanins on obesity-associated comorbidities: A concise review. Foods.

[42] Youdim K.A., Shukitt-Hale B., MacKinnon S., Kalt W., Joseph J.A. (2000). Polyphenolics enhance red blood cell resistance to oxidative stress: In vitro and in vivo. Biochim. Biophys. Acta.

[43] Sharma S., Khare P., Kumar A., Chunduri V., Kumar A., Kapoor P., Mangal P., Kondepudi K.K., Bishnoi M., Garg M. (2020). Anthocyanin-biofortified colored wheat prevents high fat diet–induced alterations in mice: Nutrigenomics studies. Mol. Nutr. Food Res..

[44] Chen Z., Wang C., Pan Y., Gao X., Chen H. (2018). Hypoglycemic and hypolipidemic effects of anthocyanins extract from black soybean seed coat in high fat diet and streptozotocin-induced diabetic mice. Food Funct..

[45] Tian X., Lu Q., Zhao S., Li J., Luo Q., Wang X., Zhang Y., Zheng N. (2021). Purple corn anthocyanin affects lipid mechanism, flavor compound profiles, and related gene expression of longissimus thoracis et lumborum muscle in goats. Animals.

[46] Silanikove N., Tiomokin D. (1992). Toxicity induced by poultry litter consumption: Effect on parameters reflecting liver function in beef cows. Anim. Prod..

[47] Kaneko J.J., Harvey J.W., Bruss M.L. (2008). Clinical Biochemistry of Domestic Animals.

[48] Peng K., Shirley D.C., Xu Z., Huang Q., McAllister T.A., Chaves A.V., Acharya S., Liu C., Wang S., Wang Y. (2016). Effect of purple prairie clover (*Dalea purpurea Vent.*) hay and its condensed tannins on growth performance, wool growth, nutrient digestibility, blood metabolites and ruminal fermentation in lambs fed total mixed rations. Anim. Feed Sci. Technol..

[49] Dijkstra J., Forbes J.M., France J., Dijkstra J., Forbes J.M., France J. (2005). Introduction. Quantitive Aspects of Ruminant Digestion and Metabolism.

